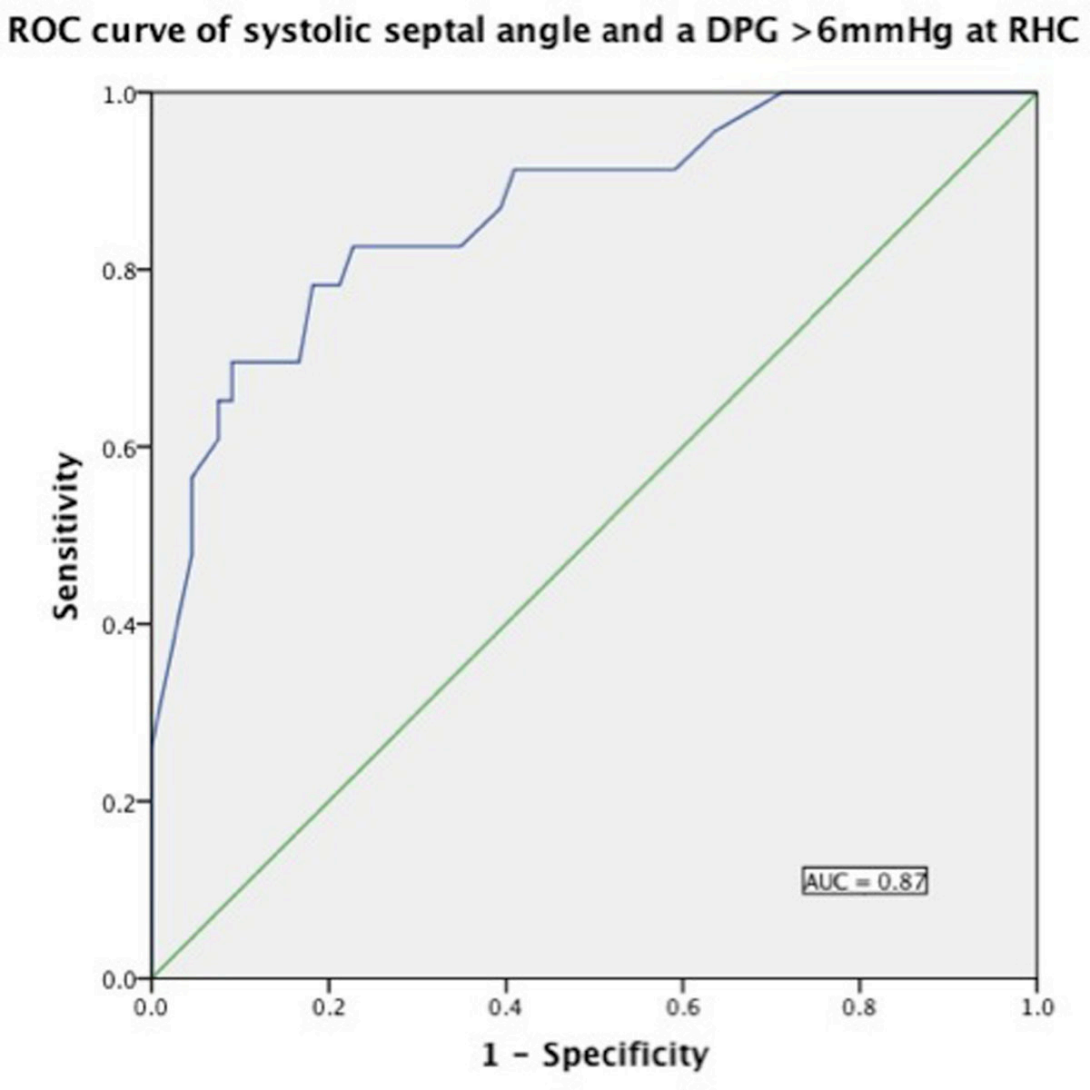# Interventricular septal angle can be used to predict which patients have combined postcapillary or precapillary pulmonary hypertension in left heart disease

**DOI:** 10.1186/1532-429X-17-S1-P338

**Published:** 2015-02-03

**Authors:** Nehal Hussain, David Capener, Charlie Elliot, Robin Condliffe, Jim M Wild, David G Kiely, Andrew Swift

**Affiliations:** 1 Sheffield Pulmonary Vascular Diseases Unit, Hallamshire Hospital, Sheffield, UK; 2 Academic Radiology, Sheffield University, Sheffield, UK

## Background

A transpulmonary gradient (TPG)>12mmHg is thought to represent evidence of vascular change beyond that expected from passive pulmonary venous congestion in patients with pulmonary hypertension and left heart disease (PH-LHD). However recent studies found those with a diastolic pressure gradient (DPG) >6 to have a worse survival. This has led to a change in the recent guidelines suggesting 2 types of PH-LHD: "isolated post-capillary PH" (pulmonary arterial wedge pressure (PAWP)>15 mm Hg and DPG<7mmHg) and "combined postcapillary PH and pre-capillary PH" (PAWP>15 mm Hg and DPG≥7 mmHg).

It would be advantageous if a non-invasive method of predicting DPG could be found for both prognostication and to identify potential patients for clinical trials of targeted therapies. Our aim was investigate the utility of cardiac magnetic resonance (CMR) imaging for estimation of DPG in patients with PH-LHD.

## Methods

Patients with suspected pulmonary hypertension underwent CMR imaging at our unit between April 2012 and April 2014. Classification followed systematic evaluation with multimodality imaging and right heart catheterisation (RHC). Patients were diagnosed as PH-LHD if no other causes of PH could be identified and they fulfilled the following criteria: signs and symptoms of heart failure; mean pulmonary artery pressure (mPAP) ≥25mmHg at rest and pulmonary arterial wedge pressure >15mmHg by RHC. TPG was defined mPAP-PAWP, and DPG was defined as diastolic PAP-PAWP.

A number of parameters were analysed including: right and left ventricular indexed volumes, ejection fractions and mass; aortic and pulmonary flow, pulmonary arterial area, and the inter-ventricular septal angle in systole and diastole.

## Results

89 patients where found to have a diagnosis of PH-LHD. The average age was 70yrs with 58% being female. Of these 89 patients, 67% were found to have a TPG>12mmHg, but only 26% had a DPG >6mmHg. No patients were found to have a raised TPG, but normal DPG. The average mPAP=44mmHg (SD±11), TPG=23mmHg (SD±11), and DPG=5 (SD±9). Systolic septal angle was the only CMR marker with a significant association with DPG, (r=0.69, p<0.0001). Receiver operating characteristics (ROC) curve of systolic septal angle against DPG>6mmHg yielded an area under the curve (AUC) of 0.87. ROC curve analysis established a systolic septal angle of >135^o^ to be the optimal threshold for distinguishing a normal DPG from those with a DPG>6mmHg (sensitivity 100%, specificity 79%). In addition linear regression was used to derive coefficients to allow formulation of an equation estimating DPG from systolic septal angle: DPG=0.359xsystolic septal angle-51.

## Conclusions

Systolic septal angle can be used to estimate whether a patient has "isolated post-capillary" or "combined postcapillary and pre-capillary" PH.

## Funding

None.

**Figure 1:**
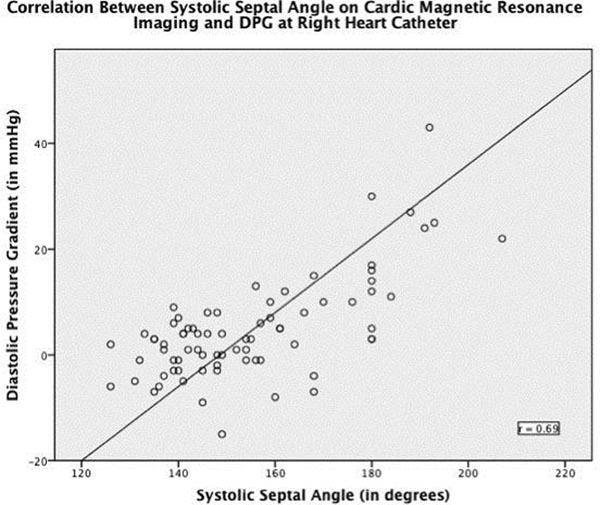


**Figure 2:**